# Operator-dependent variability of angiography-derived fractional flow reserve and the implications for treatment

**DOI:** 10.1093/ehjdh/ztab012

**Published:** 2021-02-05

**Authors:** Katherine Lal, Rebecca Gosling, Mina Ghobrial, Gareth J Williams, Vignesh Rammohan, D Rod Hose, Patricia V Lawford, Andrew Narracott, John Fenner, Julian P Gunn, Paul D Morris

**Affiliations:** 1 Department of Infection, Immunity and Cardiovascular Disease, Mathematical Modelling in Medicine Group, University of Sheffield, Beech Hill Road, Sheffield S102RX, UK; 2 Department of Cardiology, Sheffield Teaching Hospitals, NHS Foundation Trust, Sheffield, UK; 3 Insigneo Institute for In Silico Medicine, University of Sheffield, Sheffield, UK

**Keywords:** Coronary physiology, Coronary angiography, Computer modelling

## Abstract

**Aims:**

To extend the benefits of physiologically guided percutaneous coronary intervention to many more patients, angiography-derived, or ‘virtual’ fractional flow reserve (vFFR) has been developed, in which FFR is computed, based upon the images, instead of being measured invasively. The effect of operator experience with these methods upon vFFR accuracy remains unknown. We investigated variability in vFFR results based upon operator experience with image-based computational modelling techniques.

**Methods and results:**

Virtual fractional flow reserve was computed using a proprietary method (VIRTUheart) from the invasive angiograms of patients with coronary artery disease. Each case was processed by an expert (>100 vFFR cases) and a non-expert (<20 vFFR cases) operator and results were compared. The primary outcome was the variability in vFFR between experts and non-experts and the impact this had upon treatment strategy (PCI vs. conservative management). Two hundred and thirty-one vessels (199 patients) were processed. Mean non-expert and expert vFFRs were similar overall [0.76 (0.13) and 0.77 (0.16)] but there was significant variability between individual results (variability coefficient 12%, intraclass correlation coefficient 0.58), with only moderate agreement (*κ* = 0.46), and this led to a statistically significant change in management strategy in 27% of cases. Variability was significantly lower, and agreement higher, for expert operators; a change in their recommended management occurred in 10% of repeated expert measurements and 14% of inter-expert measurements.

**Conclusion:**

Virtual fractional flow reserve results are influenced by operator experience of vFFR processing. This had implications for treatment allocation. These results highlight the importance of training and quality assurance to ensure reliable, repeatable vFFR results.

## Graphical Abstract

**Figure:**
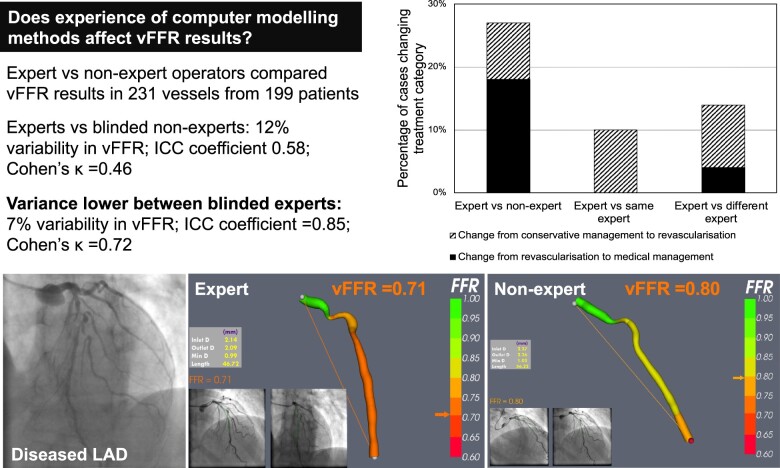

## Introduction

In the cardiac catheter laboratory, evidence robustly supports the use of physiological assessment to guide revascularization decisions. Percutaneous coronary intervention (PCI) guided by fractional flow reserve (FFR) is associated with improved clinical and economic outcomes compared to cases guided by angiography alone^1^^,^^2^ and is indicated in the major international guidelines.^3^^,^^4^ However, due to practical and methodological factors, FFR remains under-used, and thus the clinical benefits are not extended to all patients.^5^^,^^6^ Several groups have attempted to compute FFR from angiography. Different methods include quantitative flow ratio (QFR) and FFR-angiography (FFR_angio_), and are referred to collectively as virtual FFR (vFFR). Typically, vFFR is calculated by applying a mathematical solution of flow, based upon the laws of fluid dynamics, to a geometric reconstruction of coronary anatomy, derived from the angiogram. If sufficiently accurate, these models have the potential to transform interventional practice by extending the proven benefits of coronary physiological assessment to many more patients.^7^Virtual fractional flow reserve accuracy has been quantified and reported in different ways.^8^ Relative to invasive FFR, vFFR accurately identifies the binary outcome of physiological lesion significance (FFR ≤ 0.80) in around 90% of cases.^9^ This is known as ‘diagnostic’ accuracy and is heavily dependent upon the study design and patient population. Agreeability, or ‘quantitative’ accuracy of vFFR vs. invasive (measured) FFR, is less impressive, with Bland–Altman limits of agreement ±0.14 for most published models.^9^ Whereas invasive FFR is reproducible at repeated measurement,^10^ vFFR is more variable due to inconsistencies in how the arterial anatomy is reconstructed from the angiogram. This, in turn, depends upon the accuracy of the reconstruction method, and the quality of the angiogram used, how well opacified the culprit artery was and what acquisitions and projection angles were used to reconstruct the anatomy within the model. The variability introduced at this stage of vFFR is less well understood. Moreover, even when intra- and interobserver variability data are reported, it has been almost exclusively in the context of a research group, with expertise in computer modelling and familiarity with the methods they have themselves developed. Data regarding vFFR accuracy in the hands of non-experts is less well understood. This is important because some vFFR systems are now available commercially and approved for use in the cardiac catheter laboratory to make important revascularization decisions.

The aim of this study was to compare vFFR results between expert and non-expert operators and to report the impact this has upon treatment decisions.

## Methods

### Study design

This was a single-centre study composed of healthcare researchers based in Sheffield, within our research group at the University of Sheffield. Clinical data were from patients undergoing treatment at Sheffield Teaching Hospitals, NHS Foundation Trust, UK. The research was approved by the NHS research ethics committee and the institutional review board. Experts and non-experts computed vFFR. Results were compared to calculate the variability in the vFFR result between the groups. The potential impact this would have upon treatment (revascularization vs. conservative therapy) was also investigated.

### Study population and imaging protocol

Patients were included if they had a chronic or acute coronary syndrome, were being assessed for interventional therapy, and had a stenosis in at least one major epicardial artery estimated to be 40–90% lumen diameter by eye. Patients were excluded if they were <18 years of age, pregnant, had undergone previous coronary artery bypass surgery, or experienced acute ST-segment elevation myocardial infarction within 1 month. In the case of non-ST-segment elevation myocardial infarction, analysis was limited to the non-culprit arteries. Standard multiple single-plane coronary angiography was performed by the radial or femoral artery according to the operators’ standard practice. An initial screening stage was performed to exclude cases without at least two clear views of the lesion in question, at least 30° apart, with excessive table or patient movement, vessel overlap or foreshortening, or with poor contrast or absent electrocardiogram signal. Angiogram data were exported in Digital Imaging and Communications in Medicine format for vFFR analysis.

### Deriving fractional flow reserve from angiography

Virtual fractional flow reserve was computed from the angiogram using the VIRTUheart^TM^ (University of Sheffield, proprietary) model of coronary physiology. Three-dimensional (3D) coronary anatomy was reconstructed using an algebraic formula based upon the epipolar line intersections from two angiographic projections acquired ≥30° apart during end-diastole. The software incorporates a correction for between-acquisition (ventilatory or voluntary) movement by identifying points of correspondence in both projections. The resulting 3D surface mesh is a representation of the arterial lumen. This is converted to a volumetric mesh (ANSYS, PA, USA) ready for computational fluid dynamics simulation which computes the translesional pressure gradient by solving the 3D form of the Navier–Stokes equations (ANSYS, PA, USA). Virtual fractional flow reserve is reported as the ratio of the distal (Pd) to proximal (Pa) computed pressures. Further details have been published previously.^11–14^

### Expert and non-expert analysis

Experts were defined as individuals who had processed ≥100 vFFR cases, and non-experts who had processed <20 vFFR cases prior to the study. Experts and non-experts received the same training and proctoring to use the VIRTUheart^TM^ software package and used the same version of the software and method. In the context of this study, the terms expert and non-expert reflects only the level of experience of using computational modelling techniques to compute vFFR and not to expertise or experience in clinical cardiology. Operators were blinded, not only to the initial vFFR result, but also to the projection angles and frames selected by the previous operator. Thus each repeat analysis was completely blinded in all respects. The primary outcome measure was the variance between expert and non-expert operators in terms of the vFFR result and the treatment decision. The purpose of this study was to investigate the effect of operator experience on vFFR results and the implications this may have on treatment. To avoid confounding, ‘treatment decision’ (PCI vs. conservative treatment with medical therapy) was therefore, based only on whether the vFFR was ≤0.80 or not. Secondary outcome measures were the intra- and inter-expert-observer variability which was studied on a smaller cohort (*n* = 50) of cases. Finally, in cases where invasive FFR was measured as part of the clinical assessment, these data were recorded for comparison. There were five non-expert operators and four expert operators.

### Statistical analysis

Pearson’s correlation coefficient (*r*) was used to quantify linear correlation and proportion of variance (*R*^2^). Coefficient of variability (CV) was calculated, as the ratio of standard deviation (SD) and mean of repeated samples. Between group differences were displayed in Bland–Altman plots and the 95% limits of agreement were calculated as ±1.96⋅SD. For categorical data (FFR ≤ or > 0.80), Cohen’s kappa statistic (*κ*) was calculated to assess the strength of interobserver reliability. This takes into account the agreement expected by chance and is a number between −1.0 and 1.0 with values of 0; 0.10–20; 0.21–0.40; 0.41–0.60; 0.61–0.80; 0.81–0.90; and 1.0 indicating no (equivalent to chance), slight, fair, moderate, substantial, near-perfect and perfect agreement, respectively. Negative values indicate agreement worse than that expected by chance. Significance of change in indicated management was assessed using two-by-two contingency tables and McNemar’s test or paired nominal data. Continuous data are expressed as mean ± standard deviation and categorical data as a percentage, unless otherwise stated. A two-way mixed model was used to calculate the intraclass correlation coefficient (ICC) because the two observers are different but fixed and the population is random. A one-way random model was used to calculate the ICC for intra-observation because the observer was the same. To detect a ≥10% between group difference in treatment strategy (FFR < or ≥ 0.80) with 80% power at 0.05 significance level, 194 paired (expert vs. non-expert) samples were required.

## Results

### Patient and lesion characteristics

**Figure 1:**
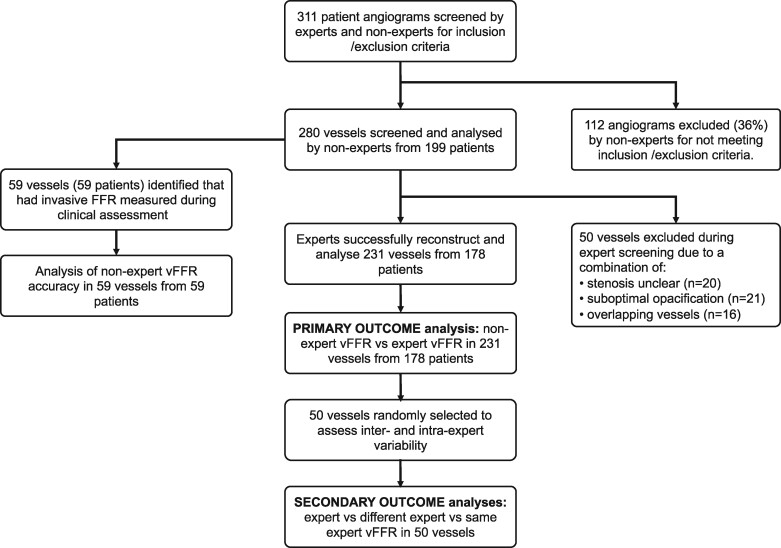
Flowchart showing patient recruitment, exclusions, and analyses.

Three hundred and eleven angiograms were screened for inclusion, and 281 (64%) from 199 patients were included and processed by the team of five non-expert operators. Of these, 50 (17.8%) were deemed unsuitable for accurate analysis by the experts (details in *Figure 1*). The remaining 231 vessels from 178 patients were successfully reconstructed and their vFFRs processed by the experts. Of these 178 patients, 61% were male, 29% were diabetic, 71% had treated hypertension, and 47% were being treated for dyslipidaemia. Fifty percent of had chronic coronary syndrome and 50% had acute coronary syndrome. Of the 231 vessels, 72% were left anterior descending (LAD), 7% were right coronary arteries (RCA), and 22% were left circumflex (LCX).

### Inter-operator variability: experts vs. non-experts

**Figure 2:**
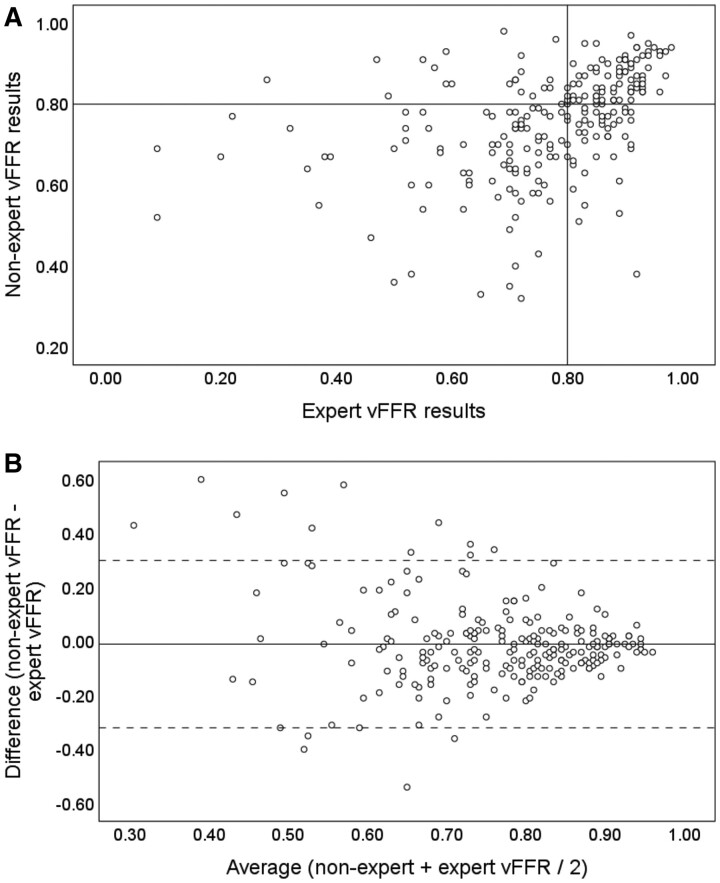
Scatter plot (*A*) and Bland–Altman plot (*B*) of non-expert vs. expert vFFR. (*A*) Correlation coefficient was 0.41 (*R*^2^ = 0.17). The horizontal and vertical line indicates the ≤0.80 FFR threshold for intervention. Cases in the upper right and lower left quadrants are concordant, whereas those in the upper left and lower right quadrants were discordant. There was a significant change in indicated treatment (PCI vs. conservative) in 27% of cases (*P* < 0.01). (*B*) The mean vFFR value is plotted (*x* axis) against the difference between the two measures (*y* axis). The solid horizontal line indicates the mean delta or ‘bias’ −0.01 (0.16). The dashed horizontal lines indicate the upper and lower limits of agreement which incorporate 95% of all observed differences (−0.32 to +0.30).

Mean non-expert vFFR was 0.76 (0.13) and mean expert vFFR was 0.77 (0.16). There was a modest but statistically significant correlation between expert and non-expert vFFR results (*r* = 0.41, *R*^2^ = 0.17, *P* < 0.01). Intraclass correlation coefficient was 0.58 [95% confidence interval (CI) 0.46–0.68, *P* < 0.01]. Coefficient of variation between experts and non-experts was 12%. After expert vFFR analysis, the indicated management was significantly different (compared with non-expert analysis) in 63 cases (27%) (*P* < 0.01). Of these, 42 (18.2%) changed from PCI to medical therapy (result changed from ≤0.80 to >0.80) and 21 (9.1%) changed from medical therapy to PCI (result changed from >0.80 to ≤0.80). Comparing between group treatment decision, Cohen’s kappa was 0.46 (*P* < 0.001) indicating only moderate agreement between expert and non-expert vFFR results. *Figure 2* demonstrates the correlation and differences between expert and non-expert vFFR results. Fifty-nine cases, the non-experts analysed had invasive FFR measurements during initial clinical assessment. In these cases, mean invasive and vFFR were 0.83 (0.09) and 0.77 (0.11). Mean delta was −0.06 (0.10) and Bland–Altman limits of agreement were −0.28 to +0.15.

### Inter observer variability: expert vs. blinded independent expert

**Figure 3:**
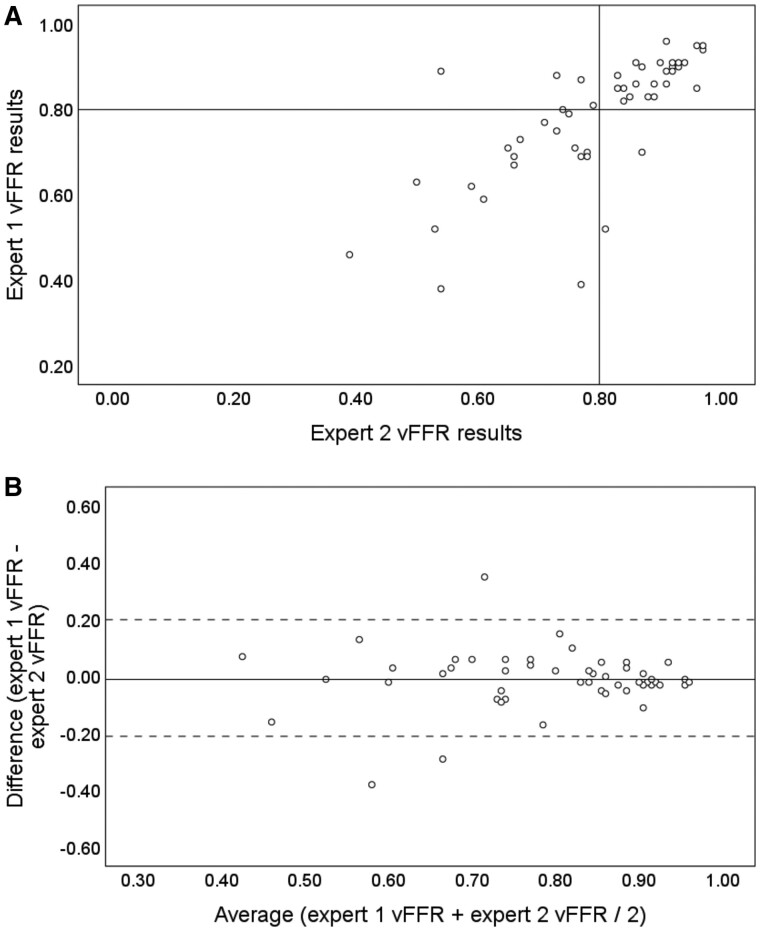
Scatter plot (*A*) and Bland–Altman plot (*B*) of expert interobserver variability. (*A*) Correlation coefficient was 0.72 (*R*^2^ = 0.52). The horizontal and vertical line indicates the ≤0.80 FFR threshold for intervention. Cases in the upper right and lower left quadrants are concordant whereas, those in the upper left and lower right quadrants were discordant, reflecting a change in indicated treatment (PCI vs. medical). About 86% were concordant and 14% were discordant. (*B*) The mean vFFR value is plotted (*x* axis) against the difference between the two measures (*y* axis). The solid horizontal line indicates the bias which was −0.01 (0.10). The dashed horizontal lines indicate the upper and lower limits of agreement which incorporate 95% of all observed differences (−0.21 to +0.20).

Fifty vessels were randomly selected for inter-expert observer analysis. To avoid bias, randomization software was used . For these cases, mean expert vFFR was 0.78 (0.15) and second expert vFFR was 0.80 (0.14). Pearson’s correlation coefficient was 0.73 (*R*^2^ 0.53, *P* < 0.01). Intraclass correlation coefficient was 0.85 (95% CI 0.73–0.91, *P* < 0.01). Coefficient of variability was 7.0%. Seven cases (14%) crossed the ≤0.80 threshold on repeat analysis; two (4%) changed from PCI to medical and five (10%) from medical to PCI. In terms of treatment decision, the Kappa statistic was 0.72 (*P* < 0.01), indicating substantial correlation, according to the pre-defined categories. Cohen’s Kappa and ICC were significantly higher than for the expert vs. non-expert analysis, and CV was significantly reduced (*P* < 0.05). Change in indicated treatment based on repeat analysis was not statistically significant (*P* > 0.05). *Figure 3* demonstrates the correlation and differences between expert and 2nd expert vFFR results.

### Intraobserver variability: expert vs. same expert

**Figure 4:**
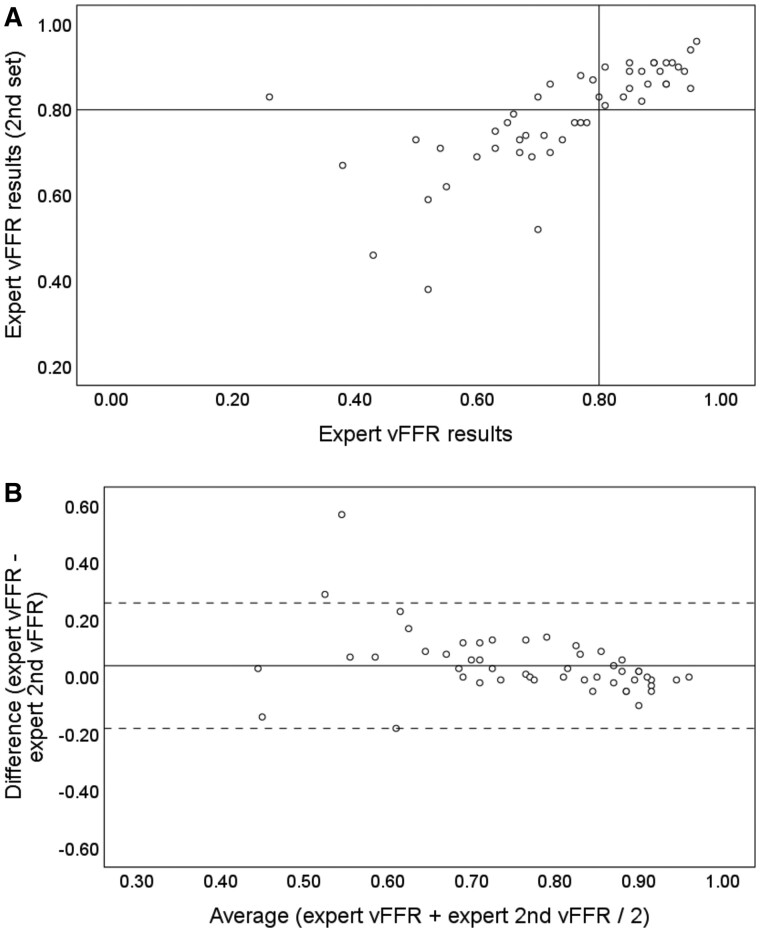
Scatter plot (*A*) and Bland–Altman plot (*B*) demonstrating expert intraobserver variability. (*A*) Correlation coefficient was 0.72 (*R*^2^ = 0.52). The horizontal and vertical line indicates the ≤0.80 FFR threshold for intervention. Cases in the upper right and lower left quadrants are concordant whereas, those in the upper left and lower right quadrants were discordant, reflecting a change in indicated treatment (PCI vs. medical). About 90% were concordant and 10% were discordant. (*B*) The mean vFFR value is plotted (*x* axis) against the difference between the two measures (*y* axis). The solid horizontal line indicates the bias which was +0.04 (0.11). The dashed horizontal lines indicate the upper and lower limits of agreement which incorporate 95% of all observed differences (−0.18 to +0.26).

**Figure 5:**
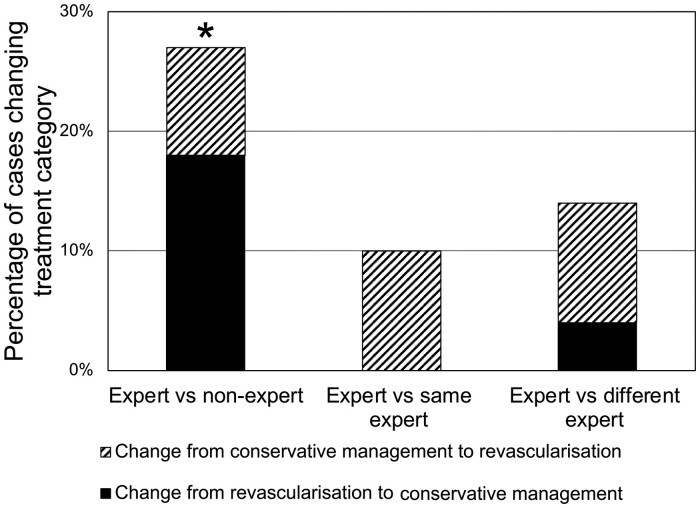
Histogram reporting the proportion of cases that changed indicated treatment strategy in each comparison. The total height reflects the percentage of all cases in which treatment was altered and the graduations reflect the nature of this change: PCI to medical therapy or medical therapy to PCI. *Statistically significant change in treatment (α = 0.01).

The same 50 cases were processed again by the same expert operator. Mean vFFR results were 0.78 (0.15) and 0.80 (0.14). The correlation coefficient was 0.72 (*R*^2^ = 0.52, *P* < 0.01). Intraclass correlation coefficient was 0.80 (95% CI 0.64–0.89, *P* < 0.01). Intra-operator coefficient of variability was 8.0%. Five cases (10%) crossed the ≤0.80 threshold on repeat analysis by the same operator, all changing from FFR medical therapy (FFR > 0.80) to PCI (FFR ≤ 0.80). In terms of treatment decision, the Kappa statistic was 0.80 (*P* < 0.01), indicating substantial correlation. Cohen’s Kappa and ICC were significantly higher than for the expert vs. non-expert analysis and CV was significantly reduced (*P* < 0.05). Change in indicated treatment based on repeat analysis was not statistically significant (*P* > 0.05). *Table 1* summarizes all statistical markers of variability for all three comparisons. *Figure 4* demonstrates the correlation and differences between expert vs. same expert vFFR results. *Figure 5* demonstrates how operator-dependent variability in vFFR analysis affected indicated treatment strategy.

**Table 1:** A summary of all statistical markers of variability and difference for all three comparisons

	Expert vs. non-expert	Expert vs. different	Expert vs. same expert
Bias (SD)	−0.01 (0.16)	−0.01 (0.10)	+0.02 (0.11)
BA limits of agreement	−0.32 to +0.30	−0.21 to +0.20	−0.18 to +0.26
Variability coefficient (95% CI)	12%^a^ (10.9–13.1%)	7% (5.6–8.4%)	8.0% (6.4–9.6%)
% change in treatment	27%^a^	14%	10%
*k* statistic	0.46^a^	0.72	0.80
Correlation coefficient	0.41^a^^,b^	0.73^b^	0.72^b^
ICC (95% CI)	0.58 (0.46–0.68)^a^	0.85 (0.73–0.91)	0.80 (0.64–0.89)

BA, Bland–Altman; ICC, intraclass correlation coefficient; k, Kappa statistic; SD, standard deviation.

a The result in column one is significantly different from those in column two and three (*α* = 0.05).

b The correlation (Pearson’s coefficient) was reached statistical significance (*α* = 0.01).

## Discussion

In this study, the largest study of interobserver variability of vFFR to date, we have demonstrated a significant difference in the results processed by experts compared with non-experts. Most importantly, this operator-dependent variability accounted for a statistically significant change in indicated treatment strategy in approximately one-quarter of cases (27%). Moreover, the kappa statistic indicated only a moderate correlation in vFFR between experts and non-experts when assigning patients to either medical therapy or PCI. Agreement was significantly stronger between experts. In this context, the kappa statistic indicated substantial correlation, and the number of cases in which a change in treatment occurred was almost half and agreement between independent experts was almost identical to that of repeated analyses by the same expert (CV 7% and 8%).

These findings are important for three main reasons. First, the accuracy of vFFR in the hands of non-experts in computer modelling has not previously been reported. Second, their results lead to a different management strategy (change between PCI and conservative) in 27% cases. Third, these models of angiography-derived vFFR are being commercialized and there is a potential risk of incorrect management being promulgated in increasing numbers of patients.

Another important finding was that non-experts processed vFFR in 18% cases that the expert subsequently deemed inadequate for analysis. This was for a number of reasons including inadequate images, poor opacification, and overlapping of vessels. This did not affect our primary outcome measure because these cases were not included in the final analysis, but it does reflect how experience of vFFR processing affects how operators assess angiogram suitability. Angiography-derived vFFR is an image-based technique that uses 2D angiographic images to reconstruct the coronary anatomy in a 3D model. The coronary arteries are particularly challenging to accurately reconstruct: they are typically just 2–3 mm in diameter; they are constantly moving with the cardiac cycle, breathing, and patient movement; there are frequently overlapping vessels which confuses the image registration software; poor opacification can cause the software to misrepresent the true anatomy; and errors are increased in regions where the epipolar lines become parallel. The findings of our study suggest that an understanding of the methodological traits and nuances, concealed behind the software’s user-interface, are important in case selection, minimizing errors, and improving repeatability. Our 17.8% exclusion rate is smaller than that reported by other methods, but that apparently modest proportion followed an initial screening process in which many more cases (36%) were excluded because of inadequate angiographic images. It has previously been shown by our group that as many as 50% of standard angiograms may not be suitable for vFFR processing.^15^ The angiograms processed in our study were acquired at a large tertiary cardiac centre, so they were clinical cases performed at the discretion of a large number of individual operators, using their normal practice, and were not performed according to a research protocol. We specifically chose this approach to ensure a realistic analysis that could be applied to everyday, real-world practice.

Although not objectively assessed in this study, the authors observed several common processing habits and errors that may have contributed to the observed variance in results. Non-experts tended to be less careful than experts when selecting and segmenting images, and could be too trusting in the semi-automatic segmentation algorithms. Experts tended to be more aware of the underlying methods and limitations of the software. They were better at selecting image pairs that optimally demonstrated the stenosis anatomy, with minimal vessel overlap and optimal contrast opacification. In addition, they were more likely to perform careful manual correction at the 2D segmentation stage to ensure the arterial edge was adequately represented by the computer model. In short, an appreciation of the underpinning methods and limitations appeared valuable in accurate case processing.

Few published studies of vFFR report observer-dependent variability. Using the QFR system, van Rosendael *et al.*^16^ reported excellent expert–expert reproducibility, with 95% limits of agreement of FFR ±0.08. However, this was in a sample of only 15 cases. With the same method but in 40 cases, expert vs. same-expert analysis using identical angiographic frames (therefore not fully blinded), yielded limits of agreement of vFFR ±0.12.^17^ In another analysis using the same method, but in a much larger cohort, repeatability between in-procedure QFR and core-laboratory QFR was reported as FFR ±0.14 and R^2^ of 0.69.^18^ It is noteworthy that, in this study, the ‘in-procedure’ (non-core-laboratory) analyses were processed by certificated staff with specific training and participating sites had to submit training datasets for approval before involvement. Using FFR_angio_, in a substudy of 25 cases, Trobs *et al.*^19^ reported the expert–expert variability with *R*^2^ of 0.77 and limits of agreement ±0.13 but, in subsequent, much larger study, Pellicano *et al.*^20^ reported expert–expert variability with *R*^2^ of 0.85 and limits of agreement ±0.08. Using an alternative method for deriving vFFR, Masdjedi *et al.*^21^ also reported impressive reproducibility with *R*^2^ 0.90 and limits of agreement ±0.05. Our study is the first to make operator-dependent variability the primary outcome measure. Furthermore, unlike previous studies, we ensured full blinding for all comparisons.

This study has implications for clinical practice, focused as it was on operator-dependent variability of vFFR, not vFFR accuracy itself, which is well described elsewhere.^7^^,^^8^ Nevertheless, the non-expert factor has to be considered in the context of accuracy. The largest analysis of vFFR accuracy to date was a Bayesian meta-analysis of thirteen studies and 1842 vessel analyses.^9^ In this analysis, relative to invasive FFR, vFFR accuracy was reported as having a Bland–Altman limit of agreement ±0.14. In the context of FFR, when the clinically important range is 0.70–0.90, an error range of ±0.14 is considerable. Our study suggests that non-expert operators introduce variability that is greater than this. Methods for computing vFFR are translating rapidly into commercial products. After appropriate technical appraisal and regulatory scrutiny, it will be important that operators receive sufficient training and proctoring to ensure the results are reliable and reproducible.

Our study demonstrates that experience and expertise with angiographic reconstruction and vFFR processing affects how cases are processed, and the vFFR result itself which in our study, was enough to move 27% of cases across the ≤0.80 threshold. Just like any new clinical method or skill, operators require training, proctoring, and experience before being deemed competent. Furthermore, (and unlike in our study) competence should not be based purely on case numbers. A potential solution would be akin to computed tomography-derived vFFR, where cases are processed remotely by experts within a core laboratory. Although acceptable for computed tomographic coronary angiography, an outpatient test, it is not apposite for on-table decision-making in the catheter laboratory. We would, therefore, advocate that vFFR systems are used to inform clinical decision-making after a programme of manufacturer-approved training and proctoring, followed by the completion of a logbook of cases that can be inspected and quality assured by an expert. In total, this should include at least 20 cases but we would avoid prescribing a precise number based only on the results of this study. Virtual fractional flow reserve results will never be 100% repeatable, no matter the operators’ experience. However, systematic errors in how cases are processed can, and therefore should, be identified and corrected under the supervision of an expert operator.

Our study had some limitations. Only one method of vFFR was used in this study. The variability and repeatability between models of vFFR were not examined. Also, the definition of what constitutes an expert and a non-expert is subjective. The definition of an expert in this study was based purely upon expertise with vFFR modelling and image-based reconstruction. The intention was to compare the results from those who, in the future, will use these tools to make important clinical decisions in the catheter laboratory. Treatment changes were not significant in the expert vs. independent expert and expert vs. same expert analyses, but the groups were considerably smaller than in the primary outcome analysis. Even accounting for reconstruction problems arising from parallel epipoloar lines in RCA cases, there were relatively few right coronary cases. However, we believe the results are applicable to both left and right coronary cases. The identification of culprit vessels was susceptible to the same problems as standard clinical practice. While this may influence vFFR accuracy relative to invasive FFR, this was not the focus of our study and has no bearing on the variance between experts and non-expert operators. Given that this was a single-centre study, we were unable to detect any institutional influence on the vFFR variance. Consistent with other studies, variance was greatest in cases with the lowest value of vFFR. Percentage concordance/discordance may over look this in some cases, but the reported Bland–Altman limits of agreement do not.^8^ Finally, accuracy relative to invasive FFR was not the subject of this study, because these were standard angiographic cases without pressure wire deployment.

## Conclusions

Angiography-derived vFFR is influenced by the operator’s experience with the methods used to derive vFFR. Given the same angiogram, non-expert operators achieved different vFFR results from expert operators. These differences accounted for a significant change in physiological classification and indicated treatment in approximately one-quarter of cases. Virtual fractional flow reserve was more reproducible in the hands of operators with more experience of using the vFFR system. This has implications for decisions regarding revascularization using vFFR as it translates from research use to the clinic. Angiography-derived vFFR may have the potential to extend the benefits of physiologically guided PCI to many more patients without the factors that limit use of invasive FFR, but operators require appropriate training, proctoring and experience in these methods to ensure reliable and repeatable results.
